# Supplementary material for: Community-based rehabilitation intervention for people with schizophrenia in Ethiopia (RISE) cluster-randomised controlled trial: An exploratory analysis of impact on food insecurity, underweight, alcohol use disorder and depressive symptoms
Source: Glob Ment Health (Camb). 2023 Oct 23;10:e70. doi: 10.1017/gmh.2023.67 (PMC10643237; doi:10.1017/gmh.2023.67)
Supplement: Asher et al. supplementary material 1 — Asher et al. supplementary material [file S2054425123000675sup001.docx]

Supplementary file 2

Table 1 RISE Exploratory outcomes at 6 and 12 months

| **Outcome** | **Facility-based care group** | **CBR plus facility-based care group** | **Minimally adjusted analysis** | | **Fully adjusted analysis** | | **Effect size (95% CI)** |
| --- | --- | --- | --- | --- | --- | --- | --- |
|  |  |  | **Mean difference or odds ratio (95% CI)^a^** | **P value** | **Mean difference or odds ratio (95% CI)^b^** | **p value** |  |
| **6 months** | **(n=60)** | **(n=52)** |  |  |  |  |  |
| **Food insecurity** | 8 (13.3%) | 4 (7.7%) | 0.89 (0.22, 3.57)^c^ | 0.87 | 0.54 (0.07, 4.04)^c^ | 0.55 | - |
| **Underweight (BMI <18.5)** (n=82) | 17 (38.6%) | 12 (31.6%) | 0.40 (0.08, 1.94) | 0.26 | 0.19 (0.02, 1.64) | 0.13 | - |
| **Alcohol use disorder (AUDIT ≥8)** (n=111) | 10 (17.0%) | 9 (17.3%) | 0.80 (0.24, 2.67) ^d^ | 0.71 | 0.95 (0.20, 4.61) ^d^ | 0.95 | - |
| **Depressive symptoms (PHQ-9 score)** | 7.1 (4.8) | 7.4 (4.0) | 0.44 (-1.32, 2.21) | 0.62 | 0.84 (-1.32, 3.00) | 0.45 | -0.19 (-0.56, 0.18) |
| **12 months** | **(n=76)** | **(n=73)** |  |  |  |  |  |
| **Food insecurity** | 6 (7.9%) | 6 (8.2%) | 0.88 (0.23, 3.36)^f^ | 0.85 | 0.37 (0.05, 3.00)^f^ | 0.35 | - |
| **Underweight (BMI <18.5) (n=122)** | 24 (37.5%) | 18 (31.0%) | 0.65 (0.25, 1.72)^g^ | 0.39 | 0.53 (0.17, 1.62)^g^ | 0.26 | - |
| **Alcohol use disorder (AUDIT ≥8)** | 13 (17.1%) | 6 (8.2%) | 0.33 (0.08, 1.27)^h^ | 0.11 | 0.62 (0.08, 4.85)^h^ | 0.65 | - |
| **Depressive symptoms (PHQ-9 score)** | 6.8 (4.4) | 6.3 (4.6) | -0.40 (-1.92, 1.11) | 0.60 | -0.57 (-2.18, 1.05) | 0.49 | 0.13 (-0.19, 0.45) |
