# Supplementary material for: Community-based rehabilitation intervention for people with schizophrenia in Ethiopia (RISE) cluster-randomised controlled trial: An exploratory analysis of impact on food insecurity, underweight, alcohol use disorder and depressive symptoms
Source: Glob Ment Health (Camb). 2023 Oct 23;10:e70. doi: 10.1017/gmh.2023.67 (PMC10643237; doi:10.1017/gmh.2023.67)
Supplement: Asher et al. supplementary material 2 — Asher et al. supplementary material [file S2054425123000675sup002.pptx]

## Slide 1
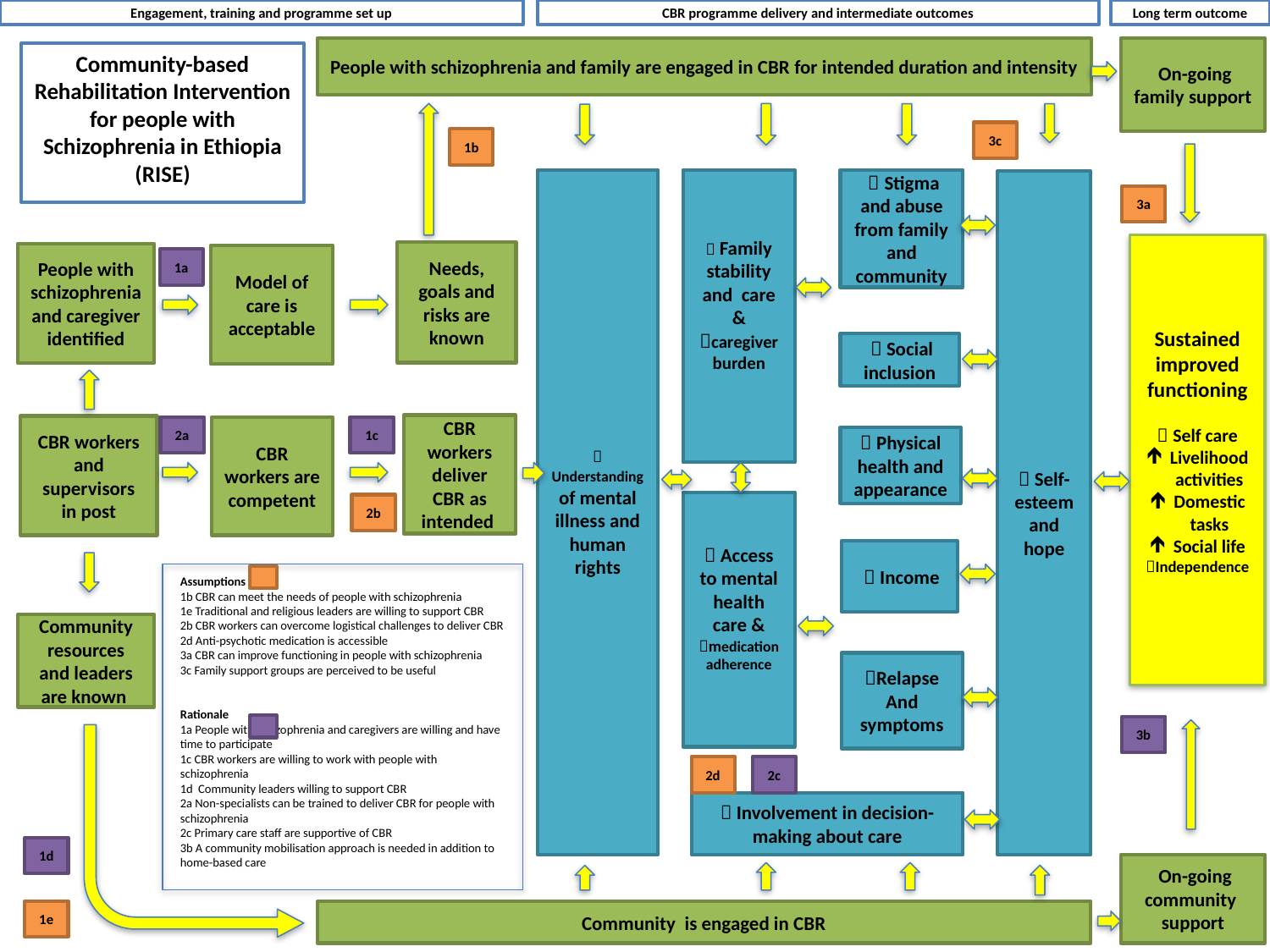

Engagement, training and programme set up
CBR programme delivery and intermediate outcomes
Long term outcome
People with schizophrenia and family are engaged in CBR for intended duration and intensity
 On-going family support
Community-based Rehabilitation Intervention for people with Schizophrenia in Ethiopia (RISE)
3c
1b
 Family stability and care &
caregiver burden
 Understanding of mental illness and human rights
  Stigma and abuse from family and community
 Self- esteem and hope
3a
Sustained improved functioning
 Self care
Livelihood activities
Domestic tasks
Social life
Independence
Needs, goals and risks are known
People with schizophrenia and caregiver identified
Model of care is acceptable
1a
  Social inclusion
CBR workers deliver CBR as intended
CBR workers and supervisors in post
2a
CBR workers are competent
1c
 Physical health and appearance
 Access to mental health care &
medication adherence
2b
  Income
Assumptions
1b CBR can meet the needs of people with schizophrenia
1e Traditional and religious leaders are willing to support CBR
2b CBR workers can overcome logistical challenges to deliver CBR
2d Anti-psychotic medication is accessible
3a CBR can improve functioning in people with schizophrenia
3c Family support groups are perceived to be useful
Rationale
1a People with schizophrenia and caregivers are willing and have time to participate
1c CBR workers are willing to work with people with schizophrenia
1d Community leaders willing to support CBR
2a Non-specialists can be trained to deliver CBR for people with schizophrenia
2c Primary care staff are supportive of CBR
3b A community mobilisation approach is needed in addition to home-based care
Community resources and leaders are known
Relapse
And symptoms
3b
2d
2c
 Involvement in decision-making about care
1d
 On-going community support
1e
Community is engaged in CBR
